# Material properties and effect of preconditioning of human sclera, optic nerve, and optic nerve sheath

**DOI:** 10.1007/s10237-021-01448-2

**Published:** 2021-04-20

**Authors:** Joseph Park, Andrew Shin, Somaye Jafari, Joseph L. Demer

**Affiliations:** 1 Department of Ophthalmology and Stein Eye Institute, University of California, Los Angeles, CA USA; 2 Department of Bioengineering, University of California, Los Angeles, CA USA; 3 Neuroscience Interdepartmental Program, University of California, Los Angeles, CA USA; 4 Intelon Optics Inc, Lexington, MA USA; 5 Stein Eye Institute, 100 Stein Plaza, UCLA, Los Angeles, CA 90095-7002 USA

**Keywords:** Human ocular tissue, Sclera, Optic nerve, Optic nerve sheath, Preconditioning, Biomechanics

## Abstract

The optic nerve (ON) is a recently recognized tractional load on the eye during larger horizontal eye rotations. In order to understand the mechanical behavior of the eye during adduction, it is necessary to characterize material properties of the sclera, ON, and in particular its sheath. We performed tensile loading of specimens taken from fresh postmortem human eyes to characterize the range of variation in their biomechanical properties and determine the effect of preconditioning. We fitted reduced polynomial hyperelastic models to represent the nonlinear tensile behavior of the anterior, equatorial, posterior, and peripapillary sclera, as well as the ON and its sheath. For comparison, we analyzed tangent moduli in low and high strain regions to represent stiffness. Scleral stiffness generally decreased from anterior to posterior ocular regions. The ON had the lowest tangent modulus, but was surrounded by a much stiffer sheath. The low-strain hyperelastic behaviors of adjacent anatomical regions of the ON, ON sheath, and posterior sclera were similar as appropriate to avoid discontinuities at their boundaries. Regional stiffnesses within individual eyes were moderately correlated, implying that mechanical properties in one region of an eye do not reliably reflect properties of another region of that eye, and that potentially pathological combinations could occur in an eye if regional properties are discrepant. Preconditioning modestly stiffened ocular tissues, except peripapillary sclera that softened. The nonlinear mechanical behavior of posterior ocular tissues permits their stresses to match closely at low strains, although progressively increasing strain causes particularly great stress in the peripapillary region.

## Introduction

The eye is a pressurized mechanical structure that is set in nearly constant rotational and translational motion within its orbital gimbal suspension by the actions of multiple extraocular muscles (Demer and Clark 2019). Moreover, the optic nerve (ON) that conveys visual signals from the eye to the brain also acts as a deforming (Chang et al. 2017; Chen et al. 2019; Suh et al. 2018) mechanical load on the moving eye (Demer 2016) and is surrounded by a sheath of cerebrospinal fluid internally loaded by communication with intracranial pressure (ICP) (Lee et al. 2020; Quigley et al. 1982; Raykin et al. 2017; Shin et al. 2017). Excessive intraocular pressure (IOP) is implicated in progressive damage to visual function in glaucoma (Grzybowski and Sak 2012). Repetitive strain injury to the eye caused by eye movements has been proposed both to be involved in ON damage in glaucoma (Demer et al. 2017; Shin et al. 2017; Sibony 2016; Wang et al. 2016), and progressive, damaging elongation and distortion of globe shape in axial myopia (Li et al. 2019). The ON is also susceptible to visually threatening damage from pathologically elevated ICP (Quigley et al. 1982; Shin et al. 2017) and may also be damaged during distention of its sheath by fluid shifts that occur in microgravity during space flight (Lee et al. 2017; Raykin et al. 2017).

A critical consideration in each of the foregoing disorders is the biomechanical behavior of the ocular and orbital tissues. Classical uniaxial tensile loading has been the most popular experimental method of characterizing ocular biomechanics (Elsheikh et al. 2010a; Friberg and Lace 1988; Wollensak and Spoerl 2004), but some investigators have characterized sclera using biaxial tensile loading and whole-globe inflation (Coudrillier et al. 2012; Eilaghi et al. 2010). The sclera has even been characterized by optomechanical birefringence (Shin et al. 2018). While there have been extensive investigations of anterior ocular tissues such as cornea (Carnell and Vito 1992; Liu and He 2009; Rahman et al. 2020), sclera (Coudrillier et al. 2013; Downs et al. 2005; Eilaghi et al. 2010; Elsheikh et al. 2010b; Pijanka et al. 2012; Wollensak and Spoerl 2004), and the lamina cribrosa (LC) of the optic disk (Brazile et al. 2018; Coudrillier et al. 2016; Feola et al. 2017; Midgett et al. 2020; Sigal et al. 2014), the biomechanical properties of tissues posterior to the globe have been comparatively neglected. Biomechanical properties of the bovine ON have been reported (Shin et al. 2017), but human ON data are currently lacking, despite the presence of abundant connective tissue around and within the human ON that distinguishes it from other neural tissue such as brain (Karim et al. 2004). The mechanical behavior of porcine ON sheath has been explored using inflation and axial loading (Raykin et al. 2017), but porcine anatomy differs substantially from the bilaminar human sheath (Le et al. 2020b) in a manner likely to alter its mechanical properties (Shin et al. 2020). It is vital to understand quantitatively the biomechanical behavior of human posterior ocular tissues such as the orbital ON and ON sheath, since these are both essential links in transmission of the sense of sight to the brain, mechanically load the globes they interact with the oculorotary muscles (Demer 2016), and may influence scleral remodeling and ocular elongation as may contribute to development and progression of myopia (Li et al. 2019).

Preconditioning is the classical process of releasing residual stress that remains in an unloaded organ (Lanir 2009; Lanir and Fung 1974) and has generally been presumed important in the characterization of biomaterials. However, the effect of preconditioning depends on the protocol and the type of tissue and may vary among tissue types (Tonge et al. 2013). Since most studies have included preconditioning as a matter of routine before any tensile loading (Cheng et al. 2009; Girard et al. 2009; Lanir 2009; Tonge et al. 2013), the significance of preconditioning for ocular tissues is currently unknown.

The current study therefore aimed in human tissue to characterize nonlinear, uniaxial tensile behavior in multiple scleral regions, in the ON, and its sheath, as well as the range of inter-individual and intraocular variation in these properties, and the degree to which properties in one region of a given eye might predict properties elsewhere in that eye. Since there have been no reported investigations of the effects of preconditioning on human ocular tissues, this study also compared tensile results with and without preconditioning in nearly identical adjacent specimens from multiple ocular regions to characterize the effects of preconditioning.

## Methods

### Specimen sources

We studied 38 pairs of fresh, unfixed human eyes, with ONs attached, collected within 3 days postmortem from four eye banks (Lions Gift of Sight, Saint Paul, MN, San Diego Eye Bank, San Diego, CA, SightLife, Seattle, WA, and OneLegacy, Los Angeles, CA). Mean donor age was 71 ± 14 (range 26–101) years, with equal numbers of males and females, all free of known ocular diseases. Eyes were obtained in conformity with the Declaration of Helsinki.

### Specimen preparation

**Fig. 1:**
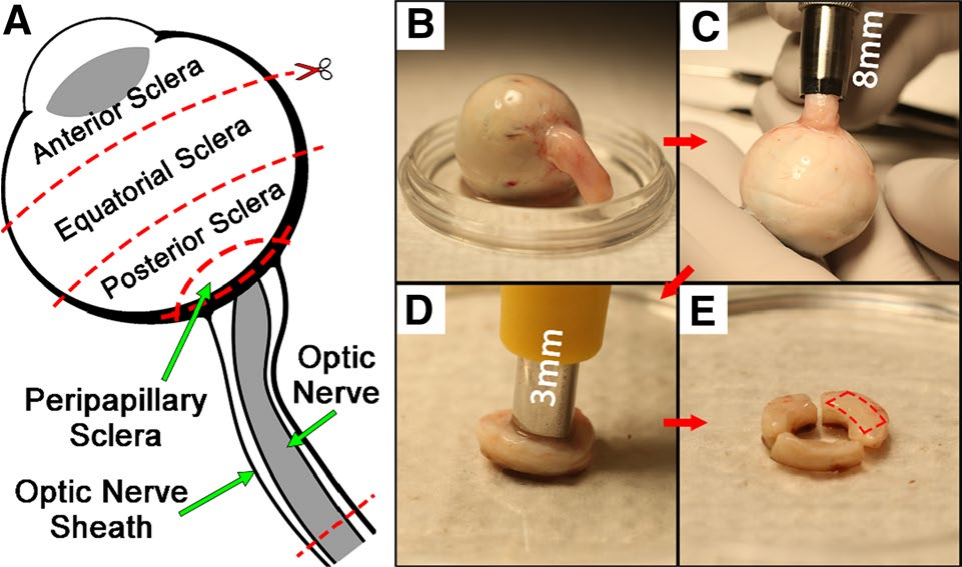
Specimen dissection. **a** Ocular tissue was divided into anterior, equatorial, posterior, and peripapillary sclera, and optic nerve and its sheath. **b** Muscles and connective tissues were excised. **c** Posterior tissues were isolated using an 8 mm inside diameter trephine. **d** The optic nerve head was removed with a 3-mm trephine. The annular peripapillary sclera was divided into thirds and trimmed into rectangular shape along the circumferential direction only due to the limited size

**Fig. 2:**
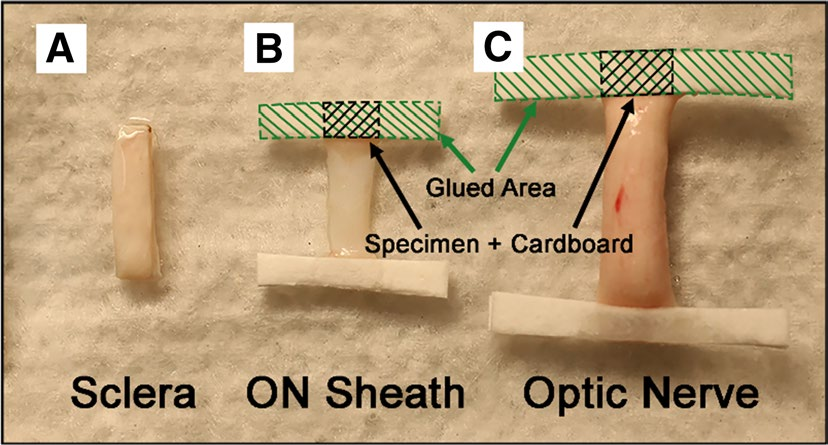
Specimen preparation. **a** The thickness of scleral tissue was enough to securely clamp on each end. **b** and **c** Cardboard was glued with cyanoacrylate to ends of the thin ON sheath and rounded ON to avoid slippage in the clamps

Specimens were stored in iced lactated Ringer’s solution during preparation and were dissected into six types: anterior, equatorial, posterior, and peripapillary sclera; ON, and ON sheath (Fig. 1a). A representative specimen is illustrated in Fig. 1b. The method of extraction of peripapillary sclera with a punch is illustrated in Fig. 1c–e. All except the cylindrical ON were trimmed into rectangular shape. Peripapillary scleral specimens were oriented with long dimensions circumferentially around the optic disk, and all other scleral tissues were taken equally oriented with long dimensions meridionally and circumferentially. Specimens of the ON sheath were oriented in both circumferential and longitudinal in equal numbers because our earlier study indicated that tensile properties of the ON sheath are similar in both orientations (Shin et al. 2020). Ends of the ON and its sheath were glued with cyanoacrylate to thin cardboard to prevent slippage (Fig. 2). Mean specimen dimensions (length × width ± standard deviation, SD) measured by digital caliper were 4.0 ± 0.6 mm × 0.8 ± 0.2 mm for anterior sclera, 4.1 ± 0.6 mm × 0.9 ± 0.2 mm for equatorial sclera, 4.0 ± 0.7 mm × 0.9 ± 0.2 mm for posterior sclera, 2.7 ± 0.8 mm × 0.8 ± 0.1 mm for peripapillary sclera, and 4.5 ± 1.3 mm × 0.8 ± 0.2 mm for ON sheath. Mean aspect ratios were 4.9, 4.7, 4.6, 3.5, and 5.6, respectively. The entire available length of the ON was untrimmed to minimize potential experimental artifact due to the thick cylindrical ends. Mean ON specimen length was 5.7 ± 2.4 mm.

### Tensile testing

Tissues were loaded uniaxially using a previously described apparatus (Shin et al. 2013, 2017) in an environmental chamber maintained by a heated water bath at 37℃ temperature and approaching 100% humidity. Initial sample length was measured using a digital caliper (Mitutoyo Co., Kawasaki, Japan). Cross-sectional area was measured using optical coherence tomography (OCT; Thorlabs Inc., Newton, NJ) after applying 0.05 N preload to eliminate slack. Loading was continuously applied at a constant rate of 0.1 mm/sec. Strain was measured based on the distance from clamp to clamp in the load cell.

### Preconditioning

**Fig. 3:**
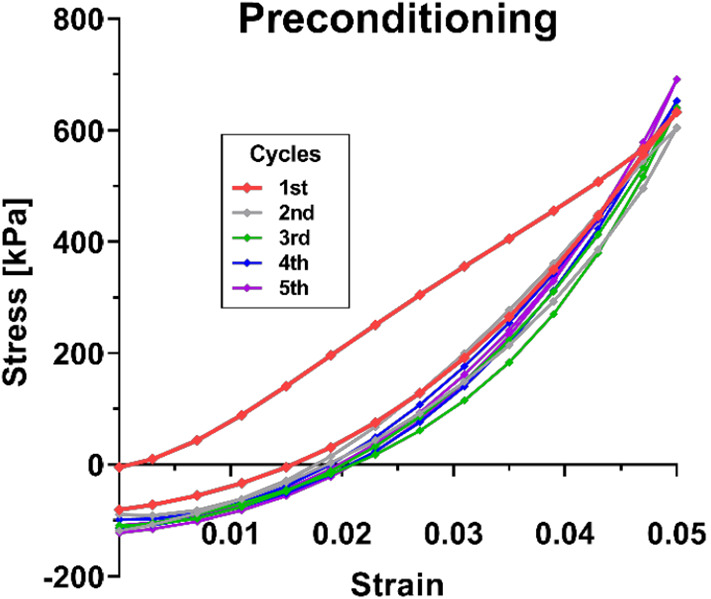
Preconditioning of posterior sclera. Stress–strain curves converged after 2 to 3 cycles of 5% cyclic loading

Specimens were treated in two fashions: approximately half of the specimens from 17 eyes underwent preconditioning and the other half did not. In 21 other eyes, no preconditioning was performed. For preconditioned specimens, we applied five cycles of tensile loading to 5% maximum strain. Figure 3 illustrates that the stress–strain curves converged to consistent responses after 2–3 loading cycles, verifying that the 5 cycles uniformly applied were sufficient to achieve stable preconditioning. Decreasing area underneath of stress–strain curves indicates release residual stress, and negative stress after the first preconditioning cycle is typical of preconditioning studies (Ajalloueian et al. 2018; Carew et al. 2000; Venkatasubramanian et al. 2006).

### Material property analysis

Force–displacement curves were converted into Cauchy stress–strain curves, to which the reduced polynomial hyperelastic models were fit using a material evaluation tool in ABAQUS 2020 (Dassault Systèmes SIMULIA Corp., Johnston, RI). This evaluation tool limits its outputs to functions that are stable in the ABAQUS numerical simulation environment for the specific empirical stress–strain curve provided. Where multiple functions were stable for a tissue, we chose the function that fits best. The strain energy density function $U$ of the reduced polynomial model is expressed by

1 $$U = \mathop \sum \limits_{i = 1}^{N} C_{i0} \left( {\overline{I}_{1} - 3} \right)^{i} + \mathop \sum \limits_{i = 1}^{N} \frac{1}{{D_{i} }}\left( {J_{el} - 1} \right)^{2i}$$

where $\overline{I}_{1}$ is the first strain invariant, $C_{i0}$ and $D_{i}$ are material constants, $J_{el}$ determines the elastic volume ratio. Depending on the stability and quality of fitting, we employed either fourth- or sixth-order reduced polynomial hyperelastic models for each tissue. For simplicity, the tissues were assumed to be isotropic and nearly incompressible (Poisson’s ratio = 0.49) to avoid singularity in computations.

## Results

### Hyperelastic properties of preconditioned tissue

**Fig. 4:**
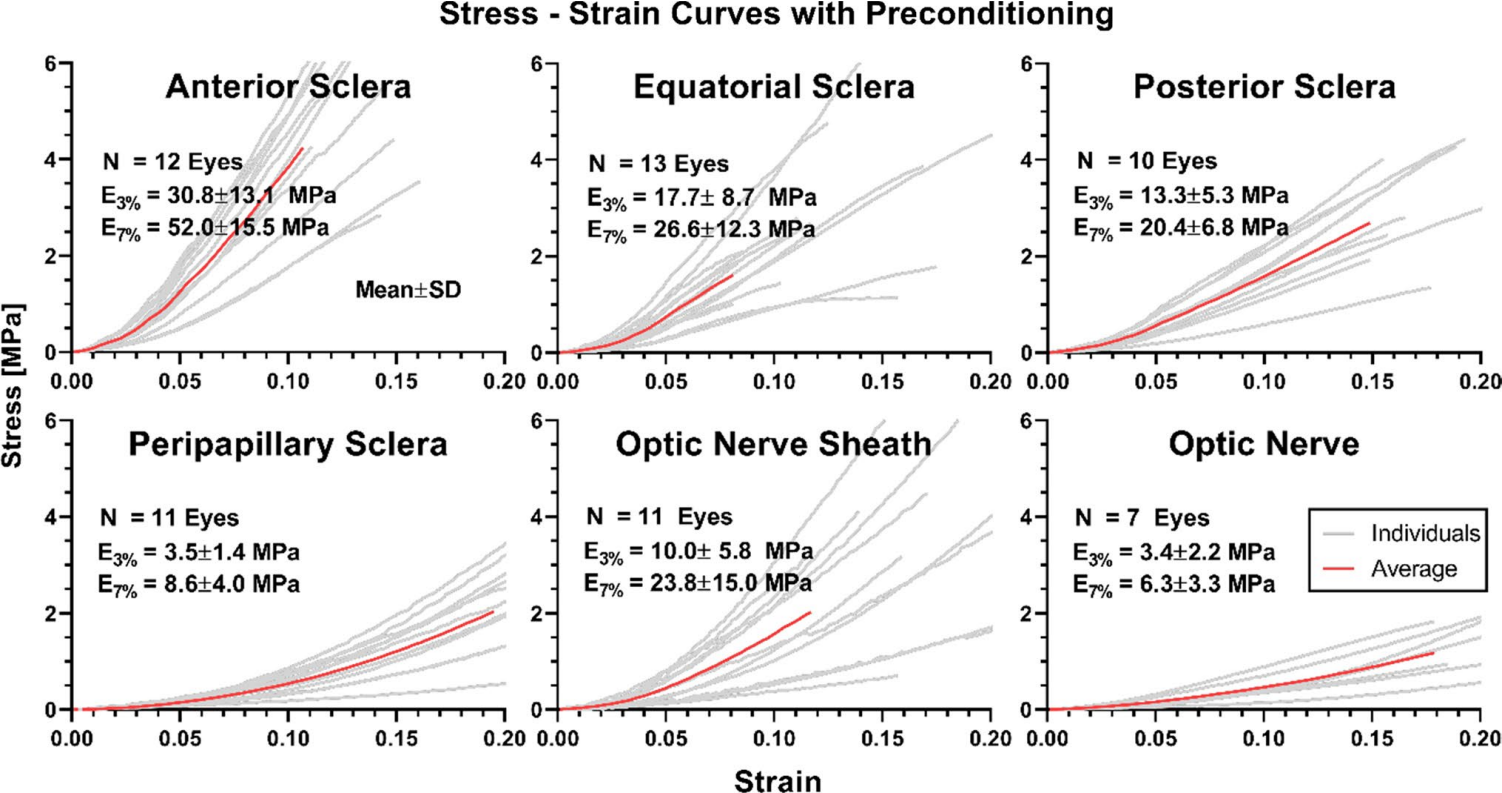
Tensile stress–strain relationships of ocular tissues with preconditioning. The E_3%_ and E_7%_ represent tangent moduli at low (3%) and high (7%) strain, respectively. Red solid curves indicate mean behaviors, and light gray lines represent individual specimens. Data are truncated to 0.20 strain for graphical clarity, although ultimate strain for peripapillary sclera was greater. N—Number of eyes

Although all tissues exhibited hyperelastic behavior as described below, it is also informative to compare tangent moduli at 3% low (*E*_3%_) and 7% high strain (*E*_7%_). Data are illustrated in Fig. 4 for specimens subjected to preconditioning. The tangent modulus at 3% strain (mean ± standard deviation, SD) of the anterior sclera was 30.8 ± 13.1 MPa, roughly twice that of equatorial and posterior sclera at 17.7 ± 8.7 MPa, and 13.3 ± 5.3 MPa, respectively. Peripapillary sclera near the ON junction, which was loaded circumferentially, had the lowest tangent modulus among all scleral tissues of 3.5 ± 1.4 MPa at 3% strain. However, because of its nonlinearity, peripapillary sclera exhibited gradual increase in tangent modulus, reaching 27.7 MPa at 30% strain. Furthermore, 8 out of 11 peripapillary scleral specimens tested had ultimate strain exceeding 34%, which is not apparent from the graph on Fig. 4 that was truncated to 20% strain for comparison with the other tissues. The tangent modulus of ON sheath at 3% strain was 10.0 ± 5.8 MPa, slightly lower than that of posterior sclera at 13.3 ± 5.4 MPa. The ON, which was loaded longitudinally, was the most compliant with 3.4 ± 2.2 MPa modulus at 3% strain.

Relative to 3% strain, the moduli at 7% strain of the anterior, equatorial, posterior, and peripapillary sclera were greater by 69% (*E*_7%_ = 52.0 ± 15.5 MPa, *P* < 0.002), 50% (*E*_7%_ = 26.6 ± 12.3 MPa, *P* = 0.044), 53% (*E*_7%_ = 20.4 ± 6.8 MPa, *P* = 0.018), and 146% (E_7%_ = 8.6 ± 4.0 MPa, *P* < 0.001), respectively (Fig. 4). At the higher strain, the ON sheath was significantly stiffer by 138% (*E*_7%_ = 23.8 ± 15.0 MPa, *P* = 0.010), and ON tangent modulus increased insignificantly by 85% (E_7%_ = 6.3 ± 3.3 MPa, *P* = 0.077).

The variability of data from individual specimens was quantified as the coefficient of variation (CV), the quotient of the SD divided by the mean value for that computed modulus. For 3% strain, the CV values were 0.43, 0.49, 0.40, and 0.40 for anterior, equatorial, posterior, and peripapillary sclera, respectively.

**Fig. 5:**
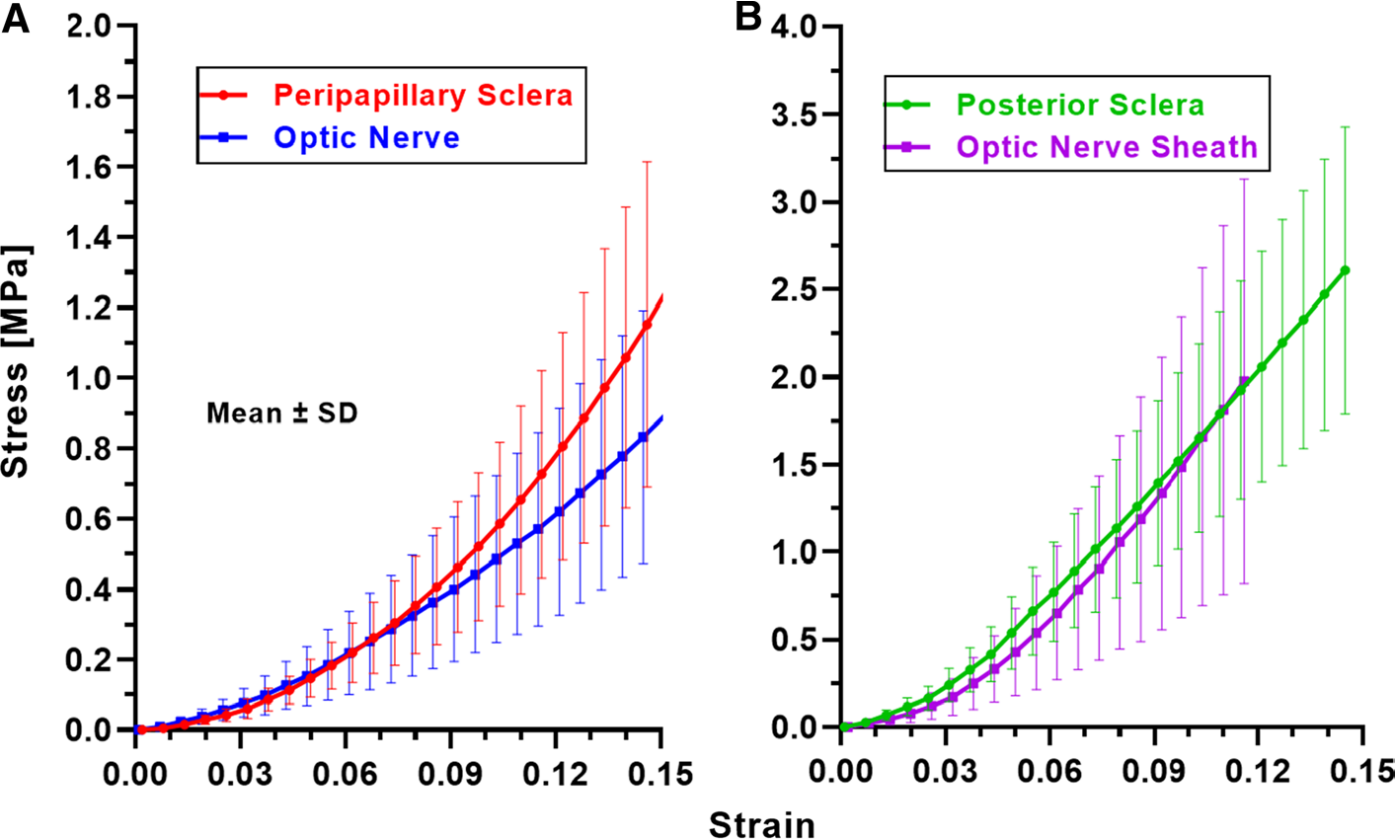
Stress–strain curves in anatomically contiguous tissues. **a** Up to about 7% strain, curves for the optic nerve and peripapillary sclera were nearly identical, but peripapillary sclera stiffened for larger strains. **b** Curves for optic nerve sheath and posterior sclera were similar throughout all strains

The forgoing data illustrate obvious nonlinear stress–strain behavior for all of the ocular tissues and regions evaluated. This nonlinearity permits tangent modulus to match between and among anatomically contiguous regions in a lower range of strains, yet be disparate outside this range. For example, Fig. 5a shows that peripapillary sclera and ON behaved nearly identical to one another up to 7% tensile strain (95% confidence intervals of polynomial and exponential fits overlapped), above which peripapillary sclera became stiffer (95% confidence intervals for linear fits differed widely). In contrast, posterior sclera and ON sheath exhibited tensile behavior similar to one another throughout the entire range of strains (95% confidence intervals of polynomial fits overlapped, Fig. 5b).

### Hyperelastic Model Parameters

Since the stress–strain curves with preconditioning were clearly nonlinear, we applied reduced polynomial hyperelastic models that fit the averaged data well (Eq. 1), using the parameters shown in Table 1. Polynomial order ranged from 4 to 6, as suitable for input into ABAQUS. Coefficients of determination (R^2^) for all six tissue regions exceeded 0.99.

**Table 1:** Reduced polynomial hyperelastic model parameters

Tissues		N	D_1_	C_10_	C_20_	C_30_	C_40_	C_50_	C_60_
Sclera	Anterior	6	0.044	0.926	512	− 42.6 × 10^3^	226 × 10^4^	− 593 × 10^5^	594 × 10^6^
	Equatorial	4	0.104	0.390	252	− 11.2 × 10^3^	219 × 10^3^	–	–
	Posterior	6	0.064	0.633	144	− 6.08 × 10^3^	153 × 10^3^	− 193 × 10^4^	955 × 10^4^
	Peripapillary	6	0.238	0.170	44.0	− 1.43 × 10^3^	27.6 × 10^3^	− 250 × 10^3^	845 × 10^3^
Optic nerve sheath	4	0.083	0.492	91.1	− 1.78 × 10^3^	16.3 × 10^3^	–	–	–
Optic nerve	6	0.000	0.268	16.0	− 143	715	− 1.73 × 10^3^	1.59 × 10^3^	–

N—polynomial degree, D_1_—[m^2^/MN], C_i0_—[MN/m^2^]

### Hyperelastic properties without preconditioning

**Fig. 6:**
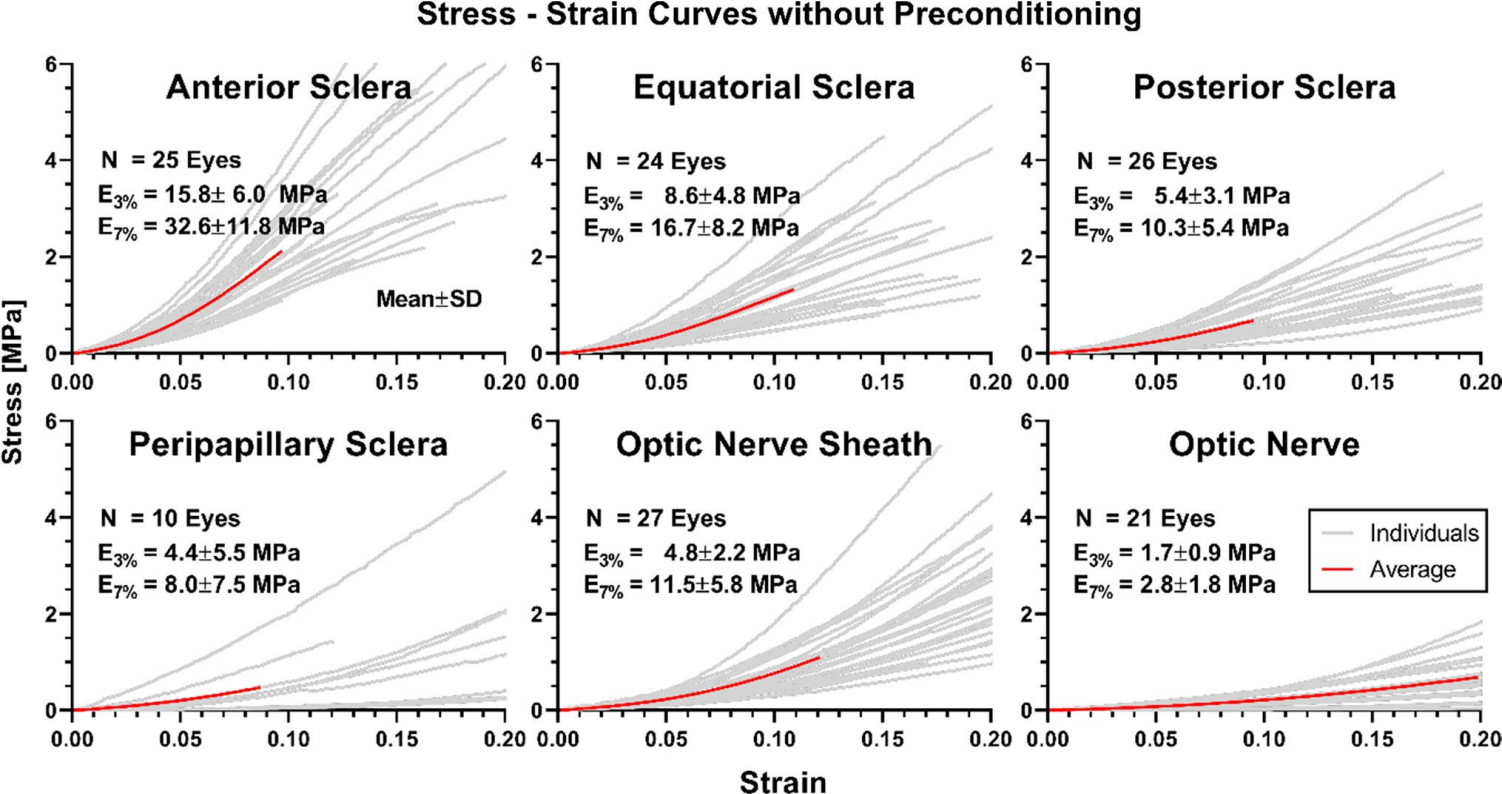
Tensile stress–strain relationships of ocular tissues without preconditioning. Conventions as in Fig. 3

Tensile data for tissues that were not preconditioned are shown in Fig. 6. These specimens were obtained from different eyes from those that underwent preconditioning. For most scleral regions and for the ON sheath, tangent modulus was significantly greater at 7% strain than at 3% strain (P < 0.001). Tangent modulus of anterior scleral modulus increased from 15.8 ± 6.0 MPa at 3% stain to 32.6 ± 11.8 MPa at 7% strain, equatorial scleral modulus increased from 8.6 ± 4.8 MPa at 3% strain to 16.7 ± 8.2 MP at 7% strain, and posterior scleral modulus increased from 5.4 ± 3.1 MPa at 3% strain to 10.3 ± 5.4 MPa at 7% strain. The tangent modulus of peripapillary sclera, however, was 4.4 ± 5.5 MPa at 3% strain, statistically similar to 8.0 ± 7.5 MPa at 7% strain (*P* = 0.237). The tangent modulus of the ON sheath was 4.8 ± 2.2 MPa at 3% strain and significantly a greater 11.5 ± 5.8 MPa at 7% strain (*P* < 0.001). The tangent modulus of the ON was 1.7 ± 0.9 MPa at 3% strain and 2.8 ± 1.8 MPa at 7% strain (*P* = 0.016).

### Paired specimen comparison for preconditioning

**Fig. 7:**
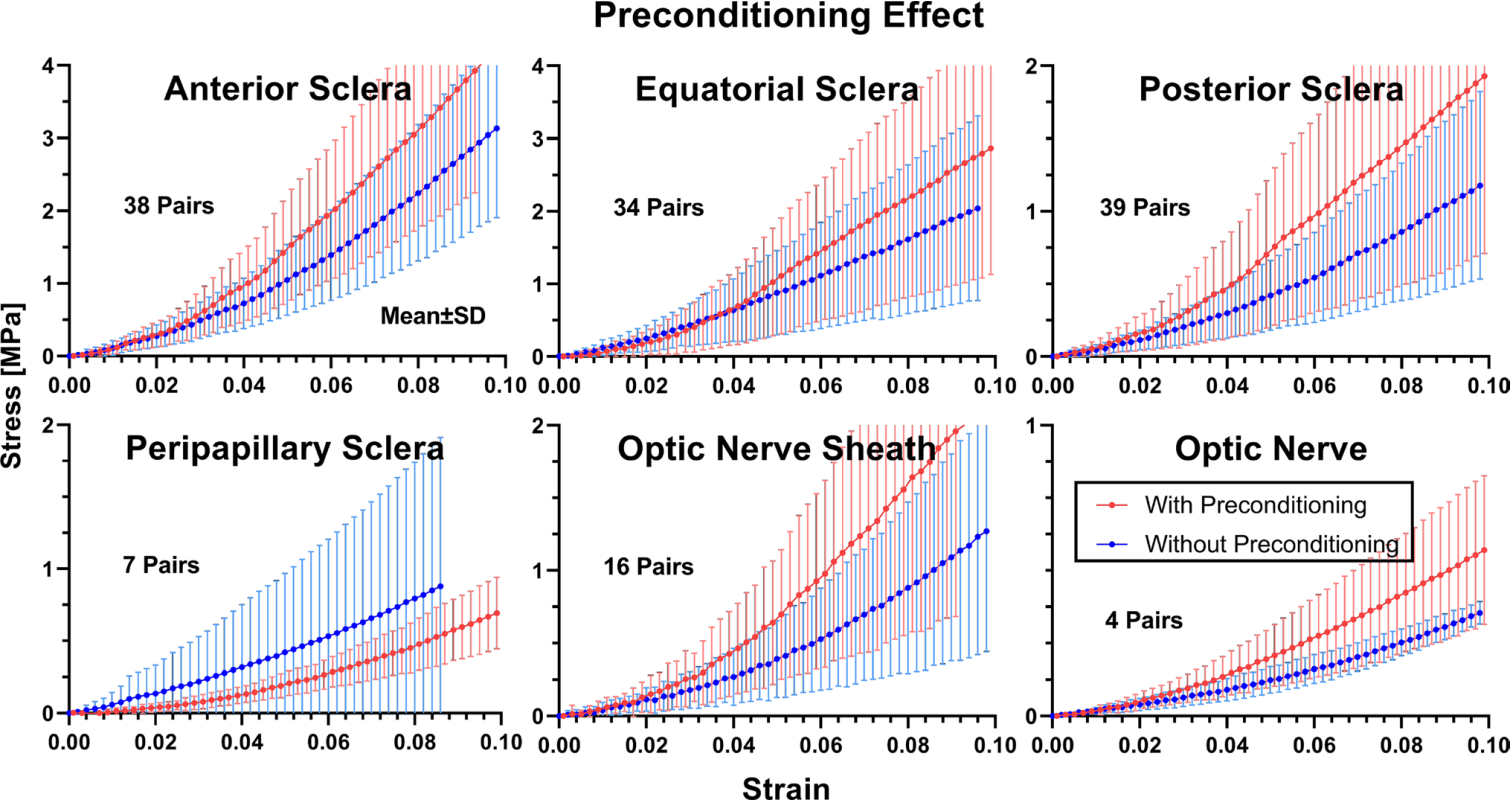
Effect of preconditioning on adjacent, nearly identical samples collected as pairs. Specimens from10 eyes were used. Error bars show data ranges, and red and blue dotted curves indicate averages

To avoid confounding by inter-eye variations, we collected multiple pairs of adjacent parallel specimens from each region of the eye; these pairs are as nearly identical as possible, except that only one of each pair was preconditioned. A special case was the ON, for which maximal length specimens from the left and right eyes of the same donor were used to secure an adequate aspect ratio for this comparison. Specimens were tested sequentially. Tensile results for specimen pairs are shown in Fig. 7. Up to 2% strain, stress–strain curves with and without preconditioning were nearly identical, except for peripapillary sclera. Above 2% strain, all tissue regions except peripapillary sclera were modestly stiffer after preconditioning, but the range of variation overlapped substantially. In contrast, peripapillary sclera did not stiffen, being insignificantly less stiff with preconditioning.

Tangent moduli at 3% and 7% strains were computed for paired specimens with and without preconditioning, and subjected to paired t testing as shown in Table 2. For anterior sclera, posterior sclera, and ON sheath, tangent moduli with preconditioning were significantly greater than without preconditioning. For peripapillary sclera and ON, these moduli were statistically similar with and without preconditioning. As shown in Fig. 7, the stress–strain curves for equatorial sclera overlap up to 4.2% strain, but diverge for greater strain. Likewise, the tangent moduli of equatorial sclera at 3% strain with and without preconditioning did not significantly differ (*P* = 0.059) but was significantly greater at 7% strain (*P* = 0.014). It should be noted, however, that the small number ON specimens available for paired testing reduced power to detect significant differences that might exist.

**Table 2:** Preconditioning effect on tangent modulus of paired specimens

		Preconditioning	Tangent Modulus at 3% Strain [MPa]				Tangent Modulus at 7% Strain [MPa]					
			N	Mean	SD	CV	T Test	N	Mean	SD	CV	T Test
Sclera	Anterior	With	38	36.0	18.4	0.51	< 0.001	37	57.1	23.7	0.42	< 0.001
		Without		23.2	10.6	0.46			42.6	17.0	0.40	
	Equatorial	With	34	25.2	18.4	0.73	0.059	33	36.5	22.4	0.61	0.014
		Without		18.8	12.6	0.67			25.6	15.9	0.62	
	Posterior	With	39	16.6	12.8	0.77	0.003	39	24.2	15.4	0.64	0.002
		Without		9.4	5.7	0.61			15.6	8.5	0.54	
	Peripapillary	With	7	4.5	1.9	0.42	0.323	7	9.3	3.7	0.40	0.451
		Without		9.0	11.4	1.27			13.0	14.0	1.08	
Optic nerve sheath		With	16	15.3	10.2	0.67	0.005	16	31.3	21.0	0.67	0.011
		Without		8.3	5.4	0.65			17.4	11.5	0.66	
Optic nerve		With	4	4.4	2.3	0.52	0.127	4	7.5	3.4	0.45	0.188
		Without		2.5	0.9	0.36			4.6	0.3	0.07	

Paired t tests compare values with and without preconditioning

CV—coefficient of variation. N—number of samples. SD—standard deviation

In Table 2 are also indicated CVs for tangent moduli of the paired tissues tested both with and without preconditioning. In general, these CV values were in the range of 0.3–0.7 for all tissues and were not appreciably influenced by preconditioning. Papillary sclera was one exception, for which CV for tangent modulus at 3% strain decreased from 1.27 without to 0.42 with preconditioning, and at 7% strain from 1.08 without to 0.40 with preconditioning. The second exception was the ON, for which CV for tangent modulus at 7% strain increased from 0.07 without to 0.45 with preconditioning.

### Regional correlations

**Fig. 8:**
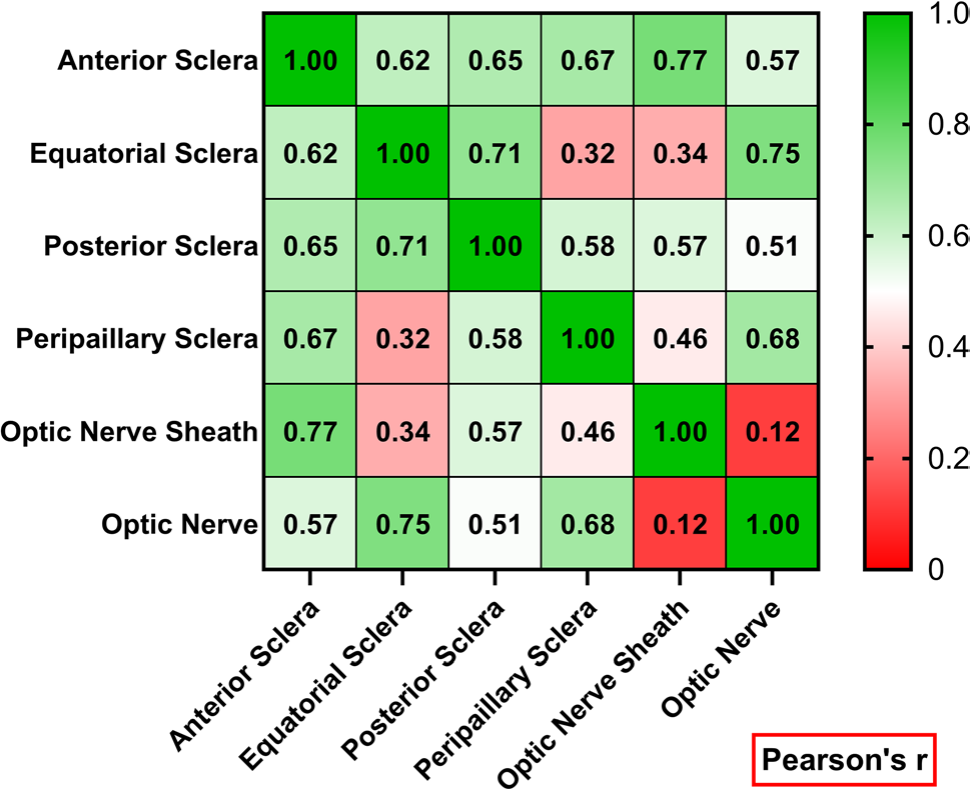
Regional cross-correlation of tangent modulus among six ocular tissue regions at 3% strain

It was of interest to determine the degree to which mechanical behavior in one region of an individual eye is reflective of behavior elsewhere in that eye. We explored this question by computing the linear correlations for 3% tangent modulus with preconditioning among all pairs of regions for all eyes. The diagonally symmetric matrix of these Pearson’s r correlations has values ranging from zero (no correlation) to one (perfect correlation, as occurs on the diagonal, Fig. 8). The correlation between ON sheath and anterior sclera was highest at 0.77, while ON sheath and ON had the smallest correlation at 0.12. Pairwise cross-correlations among scleral regions were at least 0.58, except that correlation between peripapillary and equatorial sclera was lower at 0.32.

## Discussion

The present experimental study is consistent with prior tensile characterizations of local regions of human sclera (Chen et al. 2014; Geraghty et al. 2012; Spoerl et al. 2005; Wollensak and Spoerl 2004; Woo et al. 1972), and extends by novel reduced polynomial hyperelastic models the human scleral characterizations to additionally include the ON and its sheath. This study also quantified the effect of preconditioning on these ocular tissues and demonstrated that local variation in mechanical properties by region may differ in individual eyes such that properties in one region of a given eye do not strongly reflect properties in a different region of the same eye.

### Tissue properties

Anterior sclera must be sufficiently rigid to stabilize the eye’s optics to support stable vision (Curtin 1969), and the present study confirms previous findings that the anterior sclera is appropriately stiffest of all scleral regions (Elsheikh et al. 2010a; Friberg and Lace 1988; Geraghty et al. 2012). In any given eye, scleral stiffness tended to decrease progressively in more posterior regions, so that peripapillary sclera was the most compliant. For peripapillary sclera tested here without preconditioning, the range of stress–strain curves (Fig. 4) is comparable to curves published to Spoerl et al. for this human tissue using similar conditions (Spoerl et al. 2005). The data of Spoerl et al. indicated CVs for tangent modulus at 20% strain in peripapillary sclera in the range of 0.62 ~ 0.76 (Spoerl et al. 2005), while CVs of peripapillary sclera without preconditioning from this study were 1.08 and 1.27 at 3% and 7% strains, respectively. With preconditioning, these CVs decreased to 0.42 and 0.40, at these two strains, respectively. Variability of tangent moduli in the current study is in a comparable range to the data of Spoerl et al.

Human peripapillary sclera consists of an annulus of predominantly circumferentially oriented fibers encircling the optic disk, as has been implemented in computational studies (Campbell et al. 2014; Coudrillier et al. 2013; Jones et al. 2015; Pijanka et al. 2012; Zhang et al. 2015). The current study directly evaluated tensile properties of peripapillary sclera in the circumferential direction. Although tangent modulus of peripapillary sclera in the 3% strain region was low, the nonlinear stiffness increased markedly with increasing strain, indicating that peripapillary sclera can absorb considerable deformational energy without failure. This high resistance to failure seems functionally appropriate, because peripapillary sclera functions as a reinforcing ring protecting the sensitive and compliant ON and LC (Campbell et al. 2014; Coudrillier et al. 2013; Jones et al. 2015; Pijanka et al. 2012; Zhang et al. 2015) against ON traction during eye movement (Clark et al. 2020; Demer 2016; Demer et al. 2017, 2020). It is notable that preconditioning of peripapillary sclera decreased the CV of its tangent modulus appreciably, from 1.27 to 0.42 at 3% strain, and from 1.08 to 0.40 at 7% strain. The only other comparable study of human peripapillary sclera did not employ preconditioning (Spoerl et al. 2005).

The present study obtained novel tensile data on the human ON and its sheath. The data are comparable to measurement in bovine tissue (Shin et al. 2017): the tangent modulus at 7% strain of human ON (6.3 ± 3.3 MPa) was similar to that of bovine ON (5.2 ± 0.4 MPa), although for the strain human ON sheath (23.8 ± 15.0 MPa) was about half as stiff as bovine ON sheath (44.6 ± 5.6 MPa). Tensile behavior of the human ON tested here was nearly identical to peripapillary sclera in the low strain region (Fig. 5). It would appear advantageous for material properties at the junction of ON and peripapillary sclera to be similar to minimize stress concentration at their border. Similarly, the low strain tensile behavior of the ON sheath and posterior sclera was similar to one another, which would again minimize stress concentration at their critical juncture.

Similar to the mesh-like LC that constitutes the anterior terminus of the ON (Campbell et al. 2015), the intraorbital ON also consists of a matrix of stiff intrinsic connective tissue supporting its compliant axonal components (Karim et al. 2004). Although the neural tissue presumably has very low tensile strength, the embedded connective tissue makes the ON a composite structure much stronger than its axons. Shin et al. have modeled the interwoven structure of neural and connective tissue of the bovine ON for finite element modeling using the rule of mixtures (Shin et al. 2017). The tangent modulus of the ON sheath (*E*_3%_ = 10.0 ± 5.8 MPa) is about threefold that of the ON (*E*_3%_ = 3.4 ± 2.2 MPa). The stiff ON sheath seems well suited to function as a protective element for the visually critical ON.

### Preconditioning

Preconditioning is believed to release residual stress in an unloaded organ (Cheng et al. 2009; Eshel and Lanir 2001). There have been prior studies evaluating the effects of preconditioning of bovine and porcine sclera (Tonge et al. 2013) and cornea (Tonge et al. 2013) (Boyce et al. 2007), but there has been little evaluation of the effect of preconditioning on human ocular tissues (Boyce et al. 2007; Tonge et al. 2013). As shown in Fig. 3, preconditioning both shifted the stress–strain curve downward, and increased its slope, the latter representing stiffening. Most of the stress–strain curves converged after two or three cycles of cyclic loading, indicating that the preconditioning protocol employed here achieved a steady state. The tangent moduli of all ocular tissues except peripapillary sclera increased modestly after preconditioning. Preconditioning of peripapillary sclera did not significantly change tangent modulus. It may be that the anisotropic fiber orientation of peripapillary sclera (Coudrillier et al. 2013) (Gogola et al. 2018; Jan et al. 2017; Pijanka et al. 2015) or its constant deformation during eye movements (Le et al. 2020a; Suh et al. 2017) might underlie the absence of preconditioning effect.

In the human ocular tissues studied here, the chosen preconditioning protocol did not systematically reduce variability in tensile behavior, but did increase tangent moduli modestly for all tissues except for peripapillary sclera, where there was no significant change. Biological tissues might behave differently depending on conditions of preconditioning (Kim et al. 2012; Tonge et al. 2013). While it is recognized that other preconditioning protocols (e.g., different strain levels, number of loading cycles, etc.) might have produced different results, the scarcity of fresh postmortem human ocular tissues made it impractical to systematically vary preconditioning conditions in this study.

### Regional correlation

Recognizing that linear tangent moduli incompletely represent the hyperelasticity of these tissues, we nevertheless found these linear measures of stiffness useful for statistical correlations among our many measurements in a large number of human eyes. The correlation matrix in Fig. 8 demonstrates that there is at most modest correlation in elastic properties among the various regions of individual eyes. The strongest regional correlation of 0.77 was between anterior sclera and ON sheath, which indicates only 59% of the variation in ON sheath tangent modulus is statistically attributable to variation in anterior scleral modulus. This implies that a potentially available in vivo stiffness measurement of the clinically accessible anterior sclera might on average reflect about 60% of the variation in ON sheath stiffness. Correlations of tangent moduli between other regional pairs were much lower, indicating that significant regional disparities in tissue properties variously occur in individual eyes. In general, it is not possible to accurately estimate the elastic behavior of one ocular region from the value measured in another region of the same eye. Conversely, finite element modeling studies of the biomechanical effects of phenomena such as adduction traction on the ON may reasonably presume that all possible extreme values of local stiffnesses might potentially occur in any given eye.

### Limitations

Tissue characterization was limited to uniaxial tensile behavior, only presenting computations of linearly elastic tangent moduli, at arbitrary low and high strain regions, for the reader’s convenience in statistical comparisons and comparison to published studies (Eilaghi et al. 2010; Pijanka et al. 2012). This presentation should not imply that we believe the mechanical properties actually to be linear. Further characterization and modeling of viscoelastic properties (Downs et al. 2005) is expected to be helpful to understand other biomechanical phenomena such as IOP and ON traction.

## Data Availability

Yes.

## References

[CR1] Ajalloueian F, Lemon G, Hilborn J, Chronakis IS, Fossum M (2018). Bladder biomechanics and the use of scaffolds for regenerative medicine in the urinary bladder. Nat Rev Urol.

[CR2] Boyce BL, Jones RE, Nguyen TD, Grazier JM (2007). Stress-controlled viscoelastic tensile response of bovine cornea. J Biomech.

[CR3] Brazile BL, Hua Y, Jan NJ, Wallace J, Gogola A, Sigal IA (2018). Thin lamina cribrosa beams have different collagen microstructure than thick beams. Invest Ophthalmol Vis Sci.

[CR4] Campbell IC, Coudrillier B, Ross Ethier C (2014). Biomechanics of the posterior eye: A critical role in health and disease. J Biomech Eng.

[CR5] Campbell IC, Coudrillier B, Mensah J, Abel RL, Ethier CR (2015). Automated segmentation of the lamina cribrosa using frangi's filter: A novel approach for rapid identification of tissue volume fraction and beam orientation in a trabeculated structure in the eye. J R Soc Interface.

[CR6] Carew EO, Barber JE, Vesely I (2000). Role of preconditioning and recovery time in repeated testing of aortic valve tissues: Validation through quasilinear viscoelastic theory. Ann Biomed Eng.

[CR7] Carnell PH, Vito RP (1992). A model for estimating corneal stiffness using an indenter. J Biomech Eng.

[CR8] Chang MY, Shin A, Park J, Nagiel A, Lalane RA, Schwartz SD, Demer JL (2017). Deformation of optic nerve head and peripapillary tissues by horizontal duction. Am J Ophthalmol.

[CR9] Chen K, Rowley AP, Weiland JD, Humayun MS (2014). Elastic properties of human posterior eye. J Biomed Mater Res A.

[CR10] Chen JY, Le A, De Andrade LM, Goseki T, Demer JL (2019). Compression of the choroid by horizontal duction. Invest Ophthalmol Vis Sci.

[CR11] Cheng S, Clarke EC, Bilston LE (2009). The effects of preconditioning strain on measured tissue properties. J Biomech.

[CR12] Clark RA (2020). Adduction-induced strain on the optic nerve in primary open angle glaucoma at normal intraocular pressure. Curr Eye Res.

[CR13] Coudrillier B, Tian J, Alexander S, Myers KM, Quigley HA, Nguyen TD (2012). Biomechanics of the human posterior sclera: Age- and glaucoma-related changes measured using inflation testing. Invest Ophthalmol Vis Sci.

[CR14] Coudrillier B, Boote C, Quigley HA, Nguyen TD (2013). Scleral anisotropy and its effects on the mechanical response of the optic nerve head. Biomech Model Mechanobiol.

[CR15] Coudrillier B (2016). Effects of peripapillary scleral stiffening on the deformation of the lamina cribrosa. Invest Ophthalmol Vis Sci.

[CR16] Curtin BJ (1969). Physiopathologic aspects of scleral stress-strain. Trans Am Ophthalmol Soc.

[CR17] Demer JL (2016). Optic nerve sheath as a novel mechanical load on the globe in ocular duction. Invest Ophthalmol Vis Sci.

[CR18] Demer JL, Clark RA (2019). Translation and eccentric rotation in ocular motor modeling. Prog Brain Res.

[CR19] Demer JL (2017). Magnetic resonance imaging of optic nerve traction during adduction in primary open-angle glaucoma with normal intraocular pressure. Invest Ophthalmol Vis Sci.

[CR20] Demer JL (2020). Optic nerve traction during adduction in open angle glaucoma with normal versus elevated intraocular pressure. Curr Eye Res.

[CR21] Downs JC, Suh JK, Thomas KA, Bellezza AJ, Hart RT, Burgoyne CF (2005). Viscoelastic material properties of the peripapillary sclera in normal and early-glaucoma monkey eyes. Invest Ophthalmol Vis Sci.

[CR22] Eilaghi A, Flanagan JG, Tertinegg I, Simmons CA, Wayne Brodland G, Ross Ethier C (2010). Biaxial mechanical testing of human sclera. J Biomech.

[CR23] Elsheikh A, Geraghty B, Rama P, Campanelli M, Meek KM (2010). Characterization of age-related variation in corneal biomechanical properties. J R Soc Interface.

[CR24] Elsheikh A, Geraghty B, Alhasso D, Knappett J, Campanelli M, Rama P (2010). Regional variation in the biomechanical properties of the human sclera. Exp Eye Res.

[CR25] Eshel H, Lanir Y (2001). Effects of strain level and proteoglycan depletion on preconditioning and viscoelastic responses of rat dorsal skin. Ann Biomed Eng.

[CR26] Feola AJ (2017). Deformation of the lamina cribrosa and optic nerve due to changes in cerebrospinal fluid pressure. Invest Ophthalmol Vis Sci.

[CR27] Friberg TR, Lace JW (1988). A comparison of the elastic properties of human choroid and sclera. Exp Eye Res.

[CR28] Geraghty B, Jones SW, Rama P, Akhtar R, Elsheikh A (2012). Age-related variations in the biomechanical properties of human sclera. J Mech Behav Biomed Mater.

[CR29] Girard MJ, Downs JC, Bottlang M, Burgoyne CF, Suh JK (2009). Peripapillary and posterior scleral mechanics–part ii: Experimental and inverse finite element characterization. J Biomech Eng.

[CR30] Gogola A, Jan NJ, Lathrop KL, Sigal IA (2018). Radial and circumferential collagen fibers are a feature of the peripapillary sclera of human, monkey, pig, cow, goat, and sheep. Invest Ophthalmol Vis Sci.

[CR31] Grzybowski A, Sak J (2012). The historical development of the concept of glaucoma. Acta Ophthalmol.

[CR32] Jan NJ, Lathrop K, Sigal IA (2017). Collagen architecture of the posterior pole: High-resolution wide field of view visualization and analysis using polarized light microscopy. Invest Ophthalmol Vis Sci.

[CR33] Jones HJ, Girard MJ, White N, Fautsch MP, Morgan JE, Ethier CR, Albon J (2015). Quantitative analysis of three-dimensional fibrillar collagen microstructure within the normal, aged and glaucomatous human optic nerve head. J R Soc Interface.

[CR34] Karim S, Clark RA, Poukens V, Demer JL (2004). Demonstration of systematic variation in human intraorbital optic nerve size by quantitative magnetic resonance imaging and histology. Invest Ophthalmol Vis Sci.

[CR35] Kim W, Argento A, Rozsa FW, Mallett K (2012). Constitutive behavior of ocular tissues over a range of strain rates. J Biomech Eng.

[CR36] Lanir Y (2009). Mechanisms of residual stress in soft tissues. J Biomech Eng.

[CR37] Lanir Y, Fung YC (1974). Two-dimensional mechanical properties of rabbit skin. Ii. Experimental results. J Biomech.

[CR38] Le A, Shin A, Park J, Poukens V, Demer JL (2020). Bilaminar structure of the human optic nerve sheath. Curr Eye Res.

[CR39] Le A, Chen J, Lesgart M, Gawargious BA, Suh SY, Demer JL (2020). Age-dependent deformation of the optic nerve head and peripapillary retina by horizontal duction. Am J Ophthalmol.

[CR40] Lee AG, Mader TH, Gibson CR, Tarver W (2017). Space flight-associated neuro-ocular syndrome. JAMA Ophthalmol.

[CR41] Lee C (2020). In vivo estimation of optic nerve sheath stiffness using noninvasive mri measurements and finite element modeling. J Mech Behav Biomed Mater.

[CR42] Li Y, Wei Q, Le A, Gawargious BA, Demer JL (2019). Rectus extraocular muscle paths and staphylomata in high myopia. Am J Ophthalmol.

[CR43] Liu J, He X (2009). Corneal stiffness affects iop elevation during rapid volume change in the eye. Invest Ophthalmol Vis Sci.

[CR44] Midgett DE, Jefferys JL, Quigley HA, Nguyen TD (2020). The inflation response of the human lamina cribrosa and sclera: Analysis of deformation and interaction. Acta Biomater.

[CR45] Pijanka JK (2012). Quantitative mapping of collagen fiber orientation in non-glaucoma and glaucoma posterior human sclerae. Invest Ophthalmol Vis Sci.

[CR46] Pijanka JK, Spang MT, Sorensen T, Liu J, Nguyen TD, Quigley HA, Boote C (2015). Depth-dependent changes in collagen organization in the human peripapillary sclera. PLoS ONE.

[CR47] Quigley HA, Addicks EM, Green WR (1982). Optic nerve damage in human glaucoma. Iii. Quantitative correlation of nerve fiber loss and visual field defect in glaucoma, ischemic neuropathy, papilledema, and toxic neuropathy. Arch Ophthalmol.

[CR48] Rahman N, O'Neill E, Irnaten M, Wallace D, O'Brien C (2020). Corneal stiffness and collagen cross-linking proteins in glaucoma: Potential for novel therapeutic strategy. J Ocul Pharmacol Ther.

[CR49] Raykin J (2017). Characterization of the mechanical behavior of the optic nerve sheath and its role in spaceflight-induced ophthalmic changes. Biomech Model Mechanobiol.

[CR50] Shin A, Yoo L, Demer JL (2013). Biomechanics of superior oblique z-tenotomy. J AAPOS.

[CR51] Shin A, Yoo L, Park J, Demer JL (2017). Finite element biomechanics of optic nerve sheath traction in adduction. J Biomech Eng.

[CR52] Shin A, Park J, Demer JL (2018). Opto-mechanical characterization of sclera by polarization sensitive optical coherence tomography. J Biomech.

[CR53] Shin A, Park J, Le A, Poukens V, Demer JL (2020). Bilaminar mechanics of the human optic nerve sheath. Curr Eye Res.

[CR54] Sibony PA (2016). Gaze evoked deformations of the peripapillary retina in papilledema and ischemic optic neuropathy. Invest Ophthalmol Vis Sci.

[CR55] Sigal IA, Wang B, Strouthidis NG, Akagi T, Girard MJ (2014). Recent advances in oct imaging of the lamina cribrosa. Br J Ophthalmol.

[CR56] Spoerl E, Boehm AG, Pillunat LE (2005). The influence of various substances on the biomechanical behavior of lamina cribrosa and peripapillary sclera. Invest Ophthalmol Vis Sci.

[CR57] Suh SY, Le A, Shin A, Park J, Demer JL (2017). Progressive deformation of the optic nerve head and peripapillary structures by graded horizontal duction. Invest Ophthalmol Vis Sci.

[CR58] Suh SY, Clark RA, Demer JL (2018). Optic nerve sheath tethering in adduction occurs in esotropia and hypertropia, but not in exotropia. Invest Ophthalmol Vis Sci.

[CR59] Tonge TK, Murienne BJ, Coudrillier B, Alexander S, Rothkopf W, Nguyen TD (2013). Minimal preconditioning effects observed for inflation tests of planar tissues. J Biomech Eng.

[CR60] Venkatasubramanian RT, Grassl ED, Barocas VH, Lafontaine D, Bischof JC (2006). Effects of freezing and cryopreservation on the mechanical properties of arteries. Ann Biomed Eng.

[CR61] Wang X (2016). Finite element analysis predicts large optic nerve head strains during horizontal eye movements. Invest Ophthalmol Vis Sci.

[CR62] Wollensak G, Spoerl E (2004). Collagen crosslinking of human and porcine sclera. J Cataract Refract Surg.

[CR63] Woo SL, Kobayashi AS, Schlegel WA, Lawrence C (1972). Nonlinear material properties of intact cornea and sclera. Exp Eye Res.

[CR64] Zhang L, Albon J, Jones H, Gouget CL, Ethier CR, Goh JC, Girard MJ (2015). Collagen microstructural factors influencing optic nerve head biomechanics. Invest Ophthalmol Vis Sci.

